# Strengthening health professions regulation in Cambodia: a rapid assessment

**DOI:** 10.1186/s12960-016-0104-0

**Published:** 2016-03-10

**Authors:** David Clarke, Jan Duke, Tana Wuliji, Alyson Smith, Keat Phuong, Un San

**Affiliations:** 3 Chemin des Hauts, Cornillons, Chambesy, Geneva, 1292 Switzerland; Social Workers Registration Board, 6/11 Chews Lane, Wellington, New Zealand; University Research Company, LLC, 7200 Wisconsin Avenue, Suite 600, Bethesda, MD 20814-4811 USA; University Research Company, LLC, Field Office: University of Health Sciences, Room 203, First Floor, Prime Minister Building 2, 73 Preah Monivong Boulevard, Phnom Penh, Cambodia; Cambodia Ministry of Health, #80, Samdach Penh Nouth Boulevard, Sangkat Boeung Kak 2, Khan Tuol Kork, Phnom Penh, Cambodia; Cambodia Council of Nurses, University of Health Sciences, Room 203, First Floor, Prime Minister Building 2, 73 Preah Monivong Boulevard, Phnom Penh, Cambodia

**Keywords:** Health profession regulation, Medical, Dental, Midwifery, Nursing, Pharmacy, Cambodia

## Abstract

**Background:**

This paper describes a rapid assessment of Cambodia’s current system for regulating its health professions. The assessment forms part of a co-design process to set strategic priorities for strengthening health profession regulation to improve the quality and safety of health services.

A health system approach for strengthening health professions’ regulation is underway and aims to support the Government of Cambodia’s plans for scaling up its health workforce, improving health services’ safety and quality, and meeting its Association of South East Asian Nations (ASEAN) obligations to facilitate trade in health care services.

**Methods:**

The assessment used a mixed methods approach including:

- A desktop review of key laws, plans, reports and other documents relating to the regulation of the health professions in Cambodia (medicine, dentistry, midwifery, nursing and pharmacy);
- Key informant interviews with stakeholders in Cambodia (The term “stakeholders” refers to government officials, people working on health professional regulation, people working for the various health worker training institutions and health workers at the national and provincial level);
- Surveys and questionnaires to assess Cambodian stakeholder knowledge of regulation;
- Self-assessments by members of the five Cambodian regulatory councils regarding key capacities and activities of high-performing regulatory bodies; and
- A rapid literature review to identify:
  - The key functions of health professional regulation;
  - The key issues affecting the Cambodian health sector (including relevant developments in the wider ASEAN region); and
  - “Smart” health profession regulation practices of possible relevance to Cambodia.

**Results:**

We found that the current regulatory system only partially meets Cambodia’s needs. A number of key regulatory functions are being performed, but overall, the current system was not designed with Cambodia’s specific needs in mind. The existing system is also overly complex, with considerable duplication and overlap between governance and regulatory arrangements for the five regulated professions.

**Conclusions:**

There is considerable scope for reform to the current regulatory system to better align the system to Cambodia’s:

- Current needs and circumstances;
- Health system strategic priorities; and
- International obligations.

Cambodia is also well placed to base its reformed regulatory system on recent developments of “smart regulatory practices” for health professionals.

## Background

### History of health professions regulation in Cambodia

Cambodia was a protectorate of France from 1863 and ruled as part of French Indochina until it became an independent kingdom in 1953. In the aftermath of the total destruction of the country’s institutions, infrastructure and intelligentsia caused by the Khmer Rouge rule from 1970 to 1975, Cambodia’s systems for education, health care and legislation were rebuilt based on the French system.

The impetus for establishing health profession regulation in Cambodia was primarily to address the quality of private practice health services. It was considered that the Ministry of Health provided oversight for the quality of the public sector health services including their employees. However, there was no corresponding oversight in place for private practices and the health professionals employed in these services.

The resulting Cambodian legislative framework for regulation was based on the French model of health profession regulation and resulted in the establishment of overarching laws: the “Management of private medical, paramedical and medical aid profession (2000)” and the “Law on Pharmaceutical management” (1996) and its subsequent amendment (2007). Under these laws are a number of legal instruments, including five Royal Decrees, one to establish each health profession Council, with relevant Sub-decrees for a code of ethics for each profession and *prakas* (ministerial regulations) establishing core competencies for each profession.

Under Cambodia’s legislative framework, national Councils are responsible for establishing the requirements for registration of their profession, which are then assessed and determined in the 25 provinces by local Council members for entry onto the Council’s Register. The Provincial Governor and the Provincial Health Department are responsible for issuing a licence to a private health facility based on the current registration of the health professionals providing the private services.

Cambodia’s current system thus endows the health profession Councils with two distinct roles: the role of regulating health professionals in the interest of public safety and the role of the health professional association in representing the interests of its professional members. This dual role for the Councils is based on the French regulatory model and has the potential, from time to time, to create conflicts of interest within each Council. To date, work on role separation has primarily involved establishing and funding separate health professional associations, with varying degrees of success.

### The current reform agenda

The impetus for contemporary regulatory reform comes from the fact that it is recognized by stakeholders^1^ that the current Cambodian regulatory system is no longer “fit for purpose”. The assessment of fitness for purpose involved assessing whether the current system and environment were consistent with:

- The current needs and circumstances of Cambodia’s health system;
- Contemporary approaches to health professional regulation (referred to in the study as “smart practices”);
- The Cambodian Government’s health system strategic priorities; and
- The government’s international obligations and in particular, its obligations as a member of the Association of South East Asian Nations (ASEAN).

The study concluded that the current system provided only limited scope, powers, structure and capacity for regulating health professionals in the Cambodian context, and it does not adequately deal with the regulation of health professionals working in a variety of different service settings (government, private—“for profit” and non-governmental “not for profit”) or in a mix of these settings.

This situation raises a number of difficulties:

First, there is no consistent framework for regulating all health professionals, wherever they may work.

Second, there are no effective mechanisms to assure safety and quality of the care that patients receive from all health professionals in Cambodia.

Third, the existing Councils have a limited legal ability to require and assure existing health profession standards and improve these standards over time.

Fourth, the current dual role of the Councils creates conflicts of interest that are difficult to manage.

Fifth, the current system does not meet Cambodia’s strategic objectives for its health sector in that the current system cannot adequately support the government’s objectives for improving health care quality, access to care and meeting its international obligations to its ASEAN partners in the area of health profession regulation.

### A project to strengthen the regulatory system

In February 2014, the United States Agency for International Development (USAID) Applying Science to Strengthen and Improve Systems (ASSIST) Project was asked by the USAID Mission in Cambodia to provide technical assistance to support strengthening health profession regulation.

The original request related only to the work of the Medical Council of Cambodia. Support of individual Councils, particularly medicine and midwifery, by international health development agencies was a common approach. However, this was the first initiative where the activity scope was broadened in consultation with USAID and key stakeholders. The revised scope involves a health system approach that aims to holistically strengthen the overall health professions’ regulatory system. Five independent Councils—the Medical Council of Cambodia, Dental Council of Cambodia, Cambodia Council of Midwives, Cambodian Council of Nurses and the Pharmacy Council of Cambodia—administer the current regulatory system. Together with the Ministry of Health (MOH), they seek to ensure that health professionals meet minimum standards for competence, health and professional conduct for the provision of safe, ethical and effective care.

By broadening the scope of the activity to strengthen the overall regulatory system, the project sought to address the issues of:

1. The current effectiveness of health profession regulation;
2. The authority, capacity, structure and powers of the five Councils to regulate health professions in Cambodia; and
3. Limited connections between health professions’ regulation and other existing regulatory mechanisms in the Cambodian health system.

To inform an appropriate activity design for the planned project (that would meet priority needs set by Cambodian stakeholders), the USAID ASSIST Project worked with the MOH and the five Councils to design the rapid baseline assessment discussed in this paper.

The assessment findings have subsequently been used as part of a structured consultative process to identify strengths and weaknesses and set strategic priorities for improvement of the current system. The findings of the rapid assessment and the consultative process have been used to construct a national 5-year strategy for health profession regulation.

## Methods

Our review carried out a baseline assessment of the current legal and regulatory system in Cambodia for health profession regulation using a rapid review methodology. Rapid reviews are a streamlined approach to gathering and synthesizing evidence. A rapid review methodology was adopted because the results of the review were to be used to inform the design of a work programme on health professional regulation which the Royal Government of Cambodia wished to expedite.

The purpose of this rapid review was to:

- Examine the current regulatory system and legal environment for health professions in Cambodia against mandates described in current legislation and
- Identify, in consultation with stakeholders, areas where the current regulatory system is meeting the requirements of a well-performing regulatory system (and areas where it is not).

Assess whether the current system and environment is “fit for purpose”.

“Fitness for purpose” was assessed using an adapted regulatory scorecard based on an assessment methodology developed by David Clarke and Maree Foley. The current study provided an opportunity to validate the use of this scorecard as a tool for assessing regulatory systems. The scorecard provides a rapid assessment of the current capacity and capability of regulatory systems. It uses a measurement scale of 0 to 2 with a score of:

- Zero indicating that the desired attribute is *not present*;
- One indicating that the desired attribute is *partially present*; and
- Two indicating that the desired attribute is *present*.

The scores from this analysis are then used to identify implications for possible interventions to address shortcomings in the current regulatory system.

A mixed methods approach was used for the rapid assessment carried out over a period of 3 months including:

- A desktop review of key laws, plans, reports and other documents relating to the regulation of the five health professions in Cambodia;
- Key informant interviews with stakeholders in Cambodia (at the national and provincial levels);^2^
- Development of surveys^3^ and questionnaires^4^ to assess Cambodian stakeholder knowledge of regulation; and
- The completion of a self-assessment by members of the five regulatory Councils. The self-assessment was based on the work of Benton et al. [1] who have developed a series of key indicators of measuring the performance of high-performing regulatory bodies.

One Cambodian interpreter translated the various survey instruments into Khmer and translated the results back to English. The interpreter also carried out simultaneous translations (when required) for the key informant interviews.

Finally, a rapid literature review was also undertaken to identify:

- The key functions of health profession regulation;
- The key issues affecting the Cambodian health sector (including relevant development in the wider ASEAN Region); and
- “Smart” health profession regulation practices of possible relevance to Cambodia. (We specifically looked for reviews and studies about health professional regulation from neighbouring countries to Cambodia to use as comparators for our review but were unable to find any studies.)

The main search terms used were the following: “regulation/regulated”, “health professional/health worker”, “ASEAN/mutual recognition”, “best practice” and “comparative studies”. The following databases were searched for relevant material published in English between the dates of 2000 and 2015: MEDLINE, Web of Science and Google Scholar. The literature obtained (37 publications) was then synthesized to identify whether it was possible to identify key health professional regulatory functions and to identify current international thinking on how best to approach health professional regulation.

## Results and discussion

### Cambodia’s legal framework for regulation

Our review of the current legal instruments for regulating health professions in Cambodia identified a number of challenges. The existing instruments do not apply to all health professionals and are missing many rules and controls that would be expected in a modern system for regulating health professions. For example, the existing legal instruments do not provide for adequate controls for professional practice after initial registration (no ongoing assessment of practice, no requirements for re-registration, weak complaints or non-existent complaints mechanisms, no capacity for monitoring of enforcement of standards) nor do they provide the Councils with a mandate to implement standards or assess the standards of pre-service education.

### The regulators

The system of having so many regulators (the Ministry of Health, the National Councils and up to five regional and 25 provincial Councils) and having separate legislative instruments for each profession makes the existing system complex and difficult to perform efficiently and effectively.

Although all Councils have a requirement for national-, provincial- and regional-level committees, we found that the current governance system for many of the regulators has not been fully implemented (based on an assessment of key implementation activities that remained uncompleted). For example, not all the regulatory bodies have been established at the provincial or regional levels, and the bodies that have been established are not fully functioning.

### The self-assessment

As part of the review, each Council was invited to undertake a self-assessment of performance, with each Council rating their performance as “absent”, “weak”, “adequate” or “excellent” across key elements of regulatory activity and function in four key areas:

1. Legislation advocacy and responsiveness;
2. Organizational and internal governance;
3. External governance and public accountability; and
4. Responsibilities and functions.

The subcategories and findings are presented in Table 1. In summary, four out of the five Councils rated the majority of essential elements of organizational and internal governance as being “absent” or “weak”. Three out of five Councils rated the majority of elements of external governance and public accountability as “absent” or “weak”. Two out of five Councils rated elements of responsibilities and functions as being largely “absent”. Only one Council rated most elements across the four categories of regulatory performance as being “adequate” or “excellent”.

**Table 1:** Regulatory authority self-assessment

		Councils				
		1	2	3	4	5
Legislation advocacy and responsiveness						
Implementing legislation	The regulatory body interprets legislation to facilitate and accommodate changing public protection needs	1	1	2	1	2
Advocacy	The regulatory body routinely provides comments on wide health system reform and change	0	2	1	2	1
	Promotes professional issues that are congruent with protecting the public	1	2	2	2	1
Responsiveness	The regulatory body has processes that are consistent with those of other disciplines	1	1	2	2	2
	The regulatory body keeps guidance, codes, standards, competencies and rules in step with changing expectations of the public	1	0	2	2	2
	The regulatory body interprets legislation to facilitate and accommodate changing public protection needs	0	0	2	2	2
Organizational and internal governance						
Board governance	Board Members of the regulatory body are subject to regular performance appraisal	1	0	1	0	0
	Clear criteria and the necessary competences for the selection and appointment of senior officials and board members are available	0	0	1	0	1
	Induction processes are in place for new Board Members	0	2	3	0	2
Business processes	The regulatory body collaborates with other regulatory agencies to minimize administrative burden and maximize the use and impact of data	0	0	3	0	0
	The regulatory body has mechanisms to align their accreditation systems with other agencies while continuing to fulfil their mandate	0	0	2	0	0
	Develops guidance and rules that are supportive of health system change	0	0	2	0	2
	The regulatory body uses new technology to streamline business and regulatory processes	0	0	2	0	2
	The regulatory body has mechanisms in place to detect and deal with fraudulent applications and requests for verification	0	0	3	0	1
	The regulatory body has in place disaster recovery procedures and processes	0	0	0	0	0
	The regulatory body has adequate resources to enable all responsibilities to be fully discharged	1	1	2	0	1
	Reporting lines are clear, and reports are comprehensive and timely	1	0	0	2	1
	All committees have explicit, regularly reviewed terms of reference, and the activities of the committees are reported regularly to a full regulatory body	0	0	2	2	0
Quality Improvement	The regulatory body identifies and promotes best regulatory practice	0	1	2	2	1
	The regulatory body has access to relevant expert advice to support its decision-making processes	1	0	2	0	2
	Emergent trends from the outcomes of the conduct and competence process are used to inform revisions of standards and requirements for continuing competence	0	0	2	1	0
	The regulatory body routinely examines a sample of completed continuing competence returns	0	0	1	1	0
	The regulatory body monitors its performance and seeks to continually improve the time taken to deal with fitness to practice allegations	0	0	2	1	2
External governance and public accountability						
Accountability	The regulatory body is held to account for its performance	0	1	2	1	1
	The regulatory body has a clear set of performance measures that are reported regularly	0	0	1	0	0
	While there may be multi-stakeholder input to the development of standards, codes, scopes of practice policies and procedures, their application is free of inappropriate influence by government, the profession or other interested parties	2	0	2	0	0
	The regulatory body acts in a manner that maintains the confidence of the public, professionals, employers and other key stakeholders	1	0	2	2	1
	The regulatory body has a strategic plan with linked operation objectives that are regularly reviewed and updated	2	0	1	2	0
Transparency	The regulatory body has a set of clearly defined and publically available operating procedures	1	0	2	3	1
	The regulatory body has a balance between lay and professional members	2	0	0	2	0
	The regulatory body provides clear and succinct information on their responsibilities and process to registrants and the public	1	0	3	2	1
	There are clear appeals processes that can be pursued if the decisions or the actions of the regulatory body are thought to be unsound	0	0	1	2	2
	All decision-making is transparent, documented and accessible to the profession and the public	0	0	3	3	1
Collaboration	The regulatory body engages and consults key stakeholders in the development of policy and standards	0	0	3	2	2
Responsibilities and functions						
Competence and conduct	Continuing competence procedures are in place that uses data from multiple sources	0	0	2	2	0
	The regulatory body maintains independence in resolving allegations and complaints	0	0	3	2	0
	Clear, accessible and well-publicized complaint procedures are readily available	0	0	2	0	0
	The regulatory body has standards of performance in relation to dealing with the receipt, acknowledgement, investigation and resolution of fitness to practice complaints and allegations	0	0	2	0	2
	The regulatory body has an impartial approach in dealing with an allegation both with regard to complaints and registrants	0	0	2	2	0
	An adequate range of meaningful sanctions for non-observance of the standards and non-compliance with codes of conduct is available	0	0	3	1	0
Registry integrity	The register is accurate, comprehensive and readily accessible by the public, the registrants, employers and any other interested parties	1	0	2	2	1
	The regulatory body ensures that only persons who meet stipulated criteria for licensure can practice as a health professional	2	0	3	2	1
	Registration renewal procedures are efficient and effective	0	0	3	2	0
Ethics and professional behaviour	Promotes registrant behaviour that is reflective and self-regulatory	0	0	3	2	2
	The regulatory body develops and promotes sound ethical and conduct codes	1	0	3	2	3
Standards and education	The regulatory body ensures educational programmes are aligned with the competences required by registrants for fitness to practice	0	0	3	0	1
	Professional standards and competencies are developed and set in collaboration with educational providers, employers, professional organization and the public	1	0	3	0	2
Mobility	Processes relating to health professionals wishing to migrate into or emigrate from the jurisdiction are efficient and effective	0	0	3	2	0

The 5 councils are not individually identified—they are listed as Councils 1, 2, etc. Key to scores: 3—Excellent; 2—Adequate; 1—Weak; 0—Absent

### Clarity

We found that there was considerable confusion amongst stakeholders, including the regulated professions, about key aspects of the current regulatory system.

There is a lack of clarity about whether the current legal instruments apply to professionals working in both the public and the private sectors. There is considerable inconsistency and ambiguity in the various legal instruments on this important point.

Many stakeholders did not fully understand the current legislation for health professions. As well as assessing stakeholder knowledge at the national level, we carried out a small survey of 79 health professionals (primarily doctors, some midwives and nurses) attending an education activity in one small province (Kampot). This provincial survey found that:

- Some health professionals perceive registration with Councils to be voluntary when in fact it is compulsory (75 % respondents, Kampot);
- Some health professionals believe practice is possible without registration (25 % respondents, Kampot); and
- Most health professionals felt that only registered health professionals should be allowed to provide services (77 % respondents, Kampot).

### Essential regulatory functions

To assess the performance of the Councils for the key regulatory functions, we first carried out a literature review [2, 3] and review of regulatory websites [4, 5] to identify functions that are commonly performed by regulatory councils: registration, licensing, establishing standards of professional education and practice, establishing requirements and standards for continued professional development and complaints and disciplinary functions. We also reviewed the literature for best practices in regulation [6–9].

We found that all the Cambodian Councils have a registration function established by the Royal Decree. However, of the professionals regulated by the five Councils, there were varying proportions of health professionals who were registered: 44 % physicians (public sector only), 90 % pharmacists, 66 % dentists, 66 % nurses and 88 % midwives (public sector only).

We also found that there is no current scope for licensure or renewal of registration of health professionals as a way to ensure their continued competence to practice after initial registration.

All the Councils have the role of making professional codes of ethics, and a number of Councils have a sub-decree that establishes this code. These codes are a combination of ethical principles and guidelines for practice.

The Councils do not, however, have a mandate to implement standards or assess the standards of pre-service education.

The Councils are all contributing to continuing professional development and are in the process of developing requirements that will mean professionals will need to complete continuing professional development for their ongoing registration.

None of the Councils have strong complaints and disciplinary processes. The Midwifery Council is the only Council that has established a competency framework and has developed a complaint mechanism based on this framework.

No Council has members that are not health professionals. The majority of Council members are MOH employees. The Councils have limited internal governance policies and procedures or information relating to conflict of interest.

In summary, many key functions for health professional regulation are not being performed in Cambodia. Where functions are mandated by current legislation, there are difficulties with implementation of the necessary systems and processes.

The existing regulatory system is overly complex and would require considerable resources to run effectively. There are multiple legal instruments, complex regulatory governance arrangements and considerable overlap between operations of the various Councils and the MOH.

In summary, there is considerable scope for simplification of the current system.

### Cambodia’s specific needs

We found that the current system only partially meets Cambodia’s needs. A number of important regulatory functions are being performed, but the current system was not designed based with Cambodia’s specific needs, issues and problems in mind.

The current system is not well aligned with current health system priorities. Many of the current laws predate and were not designed to meet the Cambodian Government’s strategic priorities as set out in the 2008–2015 Health Sector Strategic Plan.

We also note that the situation with the current regulatory system is likely to affect the ability of the Cambodian Government to meet its obligations to its ASEAN partners. The Royal Government of Cambodia has signed three mutual recognition agreements between ASEAN countries designed to facilitate:

- The mobility of medical practitioners, nurses and dentists within the ASEAN Region;
- Information exchange and enhanced cooperation in respect of mutual recognition of medical practitioners, dentists and nurses;
- The promotion and adoption of best practices on standards and qualifications; and
- Opportunities for capacity building and training of medical practitioners, dentists and nurses.

Any process to reform Cambodia’s health profession legislation is an opportunity to align Cambodia’s legislation with those of other ASEAN countries.

### A regulatory score card

As discussed above, part of our review involved using a regulatory scorecard to assess Cambodia’s current regulatory system for health professionals.

The scores derived from this assessment were used to identify implications for possible interventions to address short-comings in the current regulatory system.

The results of the scorecard (Table 2) show that there are many problems with the current regulatory system, indicating a need for reform (rather than minor adjustment to the current regulatory system).”

**Table 2:** Assessing Cambodia’s system for regulating health professionals

Does Cambodia have a legal/regulatory system for health professionals that assures safety and quality?			Sub total
1. Laws—maximum possible score: 4			
Are there existing laws for regulating health professionals?		1/2	1/4
Are the existing laws less than 10 years old?		0/2	
2. Regulatory institutions—maximum possible score: 6			
Is there an existing regulator(s) for health professionals?		1/2	3/6
Is the regulator(s) well resourced?		1/2	
Is the regulator running effective regulatory systems and processes?		1/2	
3. Clarity—maximum possible score: 8			
Are regulatory roles clear?		1/2	2/6
Do professionals understand their obligations?		1/2	
Is there a clear overarching strategy/policy for health professional regulation?		0/2	
4. Scope—maximum possible score: 8			
Are all health professional groups regulated?		1/2	2/8
Of the groups that are regulated, are all members regulated?	Private sector?	1/2	
	Public sector?	0/2	
	Non-governmental organization?	0/2	
5. Essential functions—maximum possible score: 10			
Are all essential health professional regulatory functions in place?	Registration requirements	1/2	4/10
	Licensing requirements	0/2	
	Standards	1/2	
	Controls on health professional education	1/2	
	Monitoring and compliance	1/2	
6. Cambodia’s needs—maximum possible score: 6			
Does the regulatory scheme address Cambodia’s current needs?		1/2	3/6
Is the current regulatory scheme contributing to the government’s broader health system objectives?		1/2	
Does the current system meet ASEAN requirements for mutual recognition of health professional qualifications?		1/2	
			15/40

## Conclusions

The rapid assessment found that there is considerable scope for reform of the current regulatory system. The USAID ASSIST Project is embarking on a 3-year project in partnership with the Councils and the Ministry of Health designed to reform and strengthen the regulatory system.

To help agree and determine priorities for reform, the USAID ASSIST Project team, the Councils and the Ministry of Health ran a 2-day National Consultation Workshop with approximately 160 representatives from key stakeholders in October 2014.

This workshop provided the opportunity to reflect on the findings of the rapid assessment and, using an interactive approach, identify the Councils’ key strategic priorities for strengthening health profession regulation in Cambodia. A further article is under development that reports on the process for running the workshop and the subsequent development of a 5-year shared strategic plan which has been developed by the Councils, the Ministry and USAID ASSIST. The Councils’ newly developed 5-year shared Strategic Plan will be used for the development of the project activity design.

## Abbreviations

ASEAN: Association of South East Asian Nations
ASSIST: Applying Science to Strengthen and Improve Systems
JICA: Japanese International Cooperation Agency
KOICA: Korea International Cooperation Agency
MOH: Ministry of Health
UNFPA: United Nations Population Fund
USAID: United States Agency for International Development
WHO: World Health Organization

## Footnotes

1 The Royal Government of Cambodia acting through the Ministry of Health, the five health professional Councils and the development partners who are working with the Cambodian Government to strengthen health professional regulation (WHO, USAID, JICA, KOICA and UNFPA).

2 Thirty-four people were interviewed on a one-on-one basis.

3 Several surveys were used; one survey involved health professionals taking part in training at the Ministry of Health, and a second survey involved people who work on health professional regulation in Cambodia. The surveys were used to ascertain participants’ awareness of Cambodia’s current requirements relating to health professional regulation.

4 All members of five Cambodia’s health professional councils were asked to complete a self assessment questionnaire.
